# Synergistic interactions of blood-borne immune cells, fibroblasts and extracellular matrix drive repair in an *in vitro* peri-implant wound healing model

**DOI:** 10.1038/srep21071

**Published:** 2016-02-17

**Authors:** Melanie A Burkhardt, Jasmin Waser, Vincent Milleret, Isabel Gerber, Maximilian Y Emmert, Jasper Foolen, Simon P Hoerstrup, Falko Schlottig, Viola Vogel

**Affiliations:** 1 Department of Health Sciences and Technology, Laboratory of Applied Mechanobiology, ETH Zurich, Zurich, 8093, Switzerland; 2 Thommen Medical AG, Grenchen, 2540, Switzerland; 3 Department of Obstetrics, Laboratory of Cell and Tissue Engineering, University Hospital Zurich, Zurich, 8091, Switzerland; 4 Swiss Center for Regenerative Medicine, Zurich, 8091, Switzerland; 5 School of Life Sciences, University of Applied Science Northwestern Switzerland, Muttenz, 4132, Switzerland

## Abstract

Low correlations of cell culture data with clinical outcomes pose major medical challenges with costly consequences. While the majority of biomaterials are tested using *in vitro* cell monocultures, the importance of synergistic interactions between different cell types on paracrine signalling has recently been highlighted. In this proof-of-concept study, we asked whether the first contact of surfaces with whole human blood could steer the tissue healing response. This hypothesis was tested using alkali-treatment of rough titanium (Ti) surfaces since they have clinically been shown to improve early implant integration and stability, yet blood-free *in vitro* cell cultures poorly correlated with *in vivo* tissue healing. We show that alkali-treatment, compared to native Ti surfaces, increased blood clot thickness, including platelet adhesion. Strikingly, blood clots with entrapped blood cells in synergistic interactions with fibroblasts, but not fibroblasts alone, upregulated the secretion of major factors associated with fast healing. This includes matrix metalloproteinases (MMPs) to break down extracellular matrix and the growth factor VEGF, known for its angiogenic potential. Consequently, *in vitro* test platforms, which consider whole blood-implant interactions, might be superior in predicting wound healing in response to biomaterial properties.

Immediately upon implantation, medical implants get exposed to the patient’s blood, which initiates the first phase of wound healing. Wound healing is a well-orchestrated process of an initial haemostasis, followed by an inflammation, tissue formation and tissue remodelling phase^1^. Initial haemostasis is a concerted process of platelet adhesion and activation, coagulation and complement activation. Upon blood contact, plasma proteins adsorb onto the implant surface^2^. Beyond blood coagulation, the physicochemical surface properties such as surface chemistry, wettability and topography of the implant material regulate complement activation and specifically adhesion and recruitment of leukocytes and platelets to the material surface or within the surface-adhering blood clot^3^,^4^,^5^. While blood clots serve the primary and tightly regulated function to stop bleeding^6^, the possibility that the presence of blood clots together with the entrapped blood-borne cells might steer healing responses though has been neglected in typical cell-based biomaterial test assays, perhaps explaining the low correlations of *in vitro* cell culture data with clinical outcomes^7^. Yet, it is well understood that communication between different cell types regulates paracrine signalling^8^,^9^.

During haemostasis platelets adhere and upon activation release a plethora of factors that regulate further coagulation and platelet activation. This includes pro- and anti-inflammatory factors, as well as chemokines and growth factors, which recruit other cells to the wound site^10^,^11^,^12^. Inflammatory reactions are regulated by the interplay of different immune cells either entrapped in the blood clot or attracted to a wound site, among others, neutrophils and monocytes, of which the latter can differentiate into macrophages. Neutrophils are present during the early wound healing stage, as they later undergo apoptosis and get phagocytosed by macrophages^13^,^14^,^15^. Phagocytic cells, i.e. neutrophils and macrophages, clean the wound site from cellular debris and pathogenic material^13^, and release inflammatory cytokines and growth factors that steer the inflammatory reaction and contribute to the formation of new tissue^4^,^16^,^17^. The interaction between the implant surface and blood components such as blood cells and fibrin(ogen) will influence the extent of blood coagulation, fibrin fibre formation and acute inflammation^18^,^19^,^20^,^21^.

During the process of early tissue formation, fibroblasts and osteogenic progenitor cells are attracted to the wound site^22^ and invade the blood clot formed on the implant surface in order to degrade the blood clot and synthesize new extracellular matrix (ECM) to restore tissue homeostasis^15^. The initial provisional fibrin matrix gets typically remodelled at the time scale of days^20^. We hypothesize here that the resulting extensive crosstalk between regulatory signalling cascades of blood-borne and invading cells together with cell-ECM interactions might dominate the healing response. The lack of such crosstalk in cell monocultures might thus be responsible for the low correlation between standard cell culture studies and clinical outcomes. This hypothesis is supported by findings that differences in the architecture and properties of blood clots can indeed affect the behaviour of infiltrating cells, as shown so far for human osteoblasts *in vitro*^23^, and that the extent and overall success of a medical implant might be directly influenced by the physicochemical properties of the implant surface^17^,^18^,^24^.

To address this hypothesis, we exploited Ti surfaces as model system, since alkali-treatment of rough Ti surfaces, which renders the surfaces superhydrophilic, has been shown to positively affect early implant integration/stability in animal studies^25^,^26^,^27^,^28^,^29^. In human patients with poorly mineralized bone, alkali-treated Ti implants were successfully loaded 8 weeks post-implantation and 97% of implants were still functional after one year^30^. Altogether, these previous findings suggest a stronger osseointegrative potential of alkali-treated Ti implants compared to native Ti implants. Additionally, alkali-treatment of Ti was shown *in vitro* to increase coagulation and platelet activation, as well as thickness and morphological composition of the surface-adhering blood clot upon blood-material interaction compared to native Ti^31^. Primary human bone cells (HBCs) showed an increased attachment on hydrophilic Ti surfaces presenting a thick blood clot and interaction of HBCs with blood clots promoted increased expression of osteogenic marker proteins alkaline phosphatase and collagen type I^23^.

Since fibroblasts are the most abundant cell type that infiltrates into blood clots in early wound healing stages and initiates the remodelling of the first provisional ECM into granulation tissue, rich in fibronectin (Fn) and collagen, we tested the hypothesis whether the presence of a blood clot can accelerate remodelling and assembly of the first *de novo* ECM and thus promote fast healing. In this proof-of-concept study and with a focus on early events, clinically used dental implant surfaces, namely sandblasted and acid-etched Ti surfaces, native or alkali-treated, were exposed to human whole blood from healthy patients, to fibroblasts or to a co-culture of whole blood with subsequently seeded fibroblasts. In a previous study with the same Ti surfaces, blood clot formation was shown to be dominated by the surface chemistry (native vs. alkali-treated) and not by surface roughness, as compared to smooth surfaces^31^; hence only rough, sandblasted and acid-etched surfaces were used in the current study. For all conditions, cell adhesion, ECM deposition and remodelling were analysed. Cellular and ECM compositions were evaluated by immunofluorescent staining against F-actin, platelet integrin αIIb (CD41), Fn and fibrin. Further, ECM remodelling and collagen production were assessed by quantifying soluble MMPs and c-terminal peptide of collagen type I. Pro-inflammatory cytokines TNFα and Il-1β as well as growth factors TGF-β1 and VEGF were measured in supernatants using commercially available ELISAs. We finally assessed whether this *in vitro* model that includes the contribution of blood-borne ECM and immune cells might be better suited as a test platform with improved clinical predictive power.

## Results

To mimic most closely a surgical situation where an implant material gets into contact with the patient’s blood, this study used fresh human whole blood from 8 healthy patients (23–35 years old) within 2 hours after it had been drawn. Ti surfaces were exposed to blood for 2 hours in rotating Teflon chambers under exclusion from air, so blood coagulation was initiated upon blood-material contact. After washing off non-adhering blood components the samples were placed into cell culture medium and then seeded with fibroblasts. Only human serum from blood type AB, free of blood type specific A and B iso-agglutinins, was employed in the cell culture medium to eliminate blood type dependent immune responses. The experimental findings of this study are based on experiments done with blood from healthy donors, not including blood from patients with wound healing diseases, and co-culturing those with primary fibroblasts from allogeneic origin. For experimental reasons, blood samples were anti-coagulated with heparin at a low concentration, possibly reducing or slowing down coagulation reactions.

### Alkali-treatment of Ti surfaces enhanced blood clotting and the number of adhering blood cells

**Figure 1:**
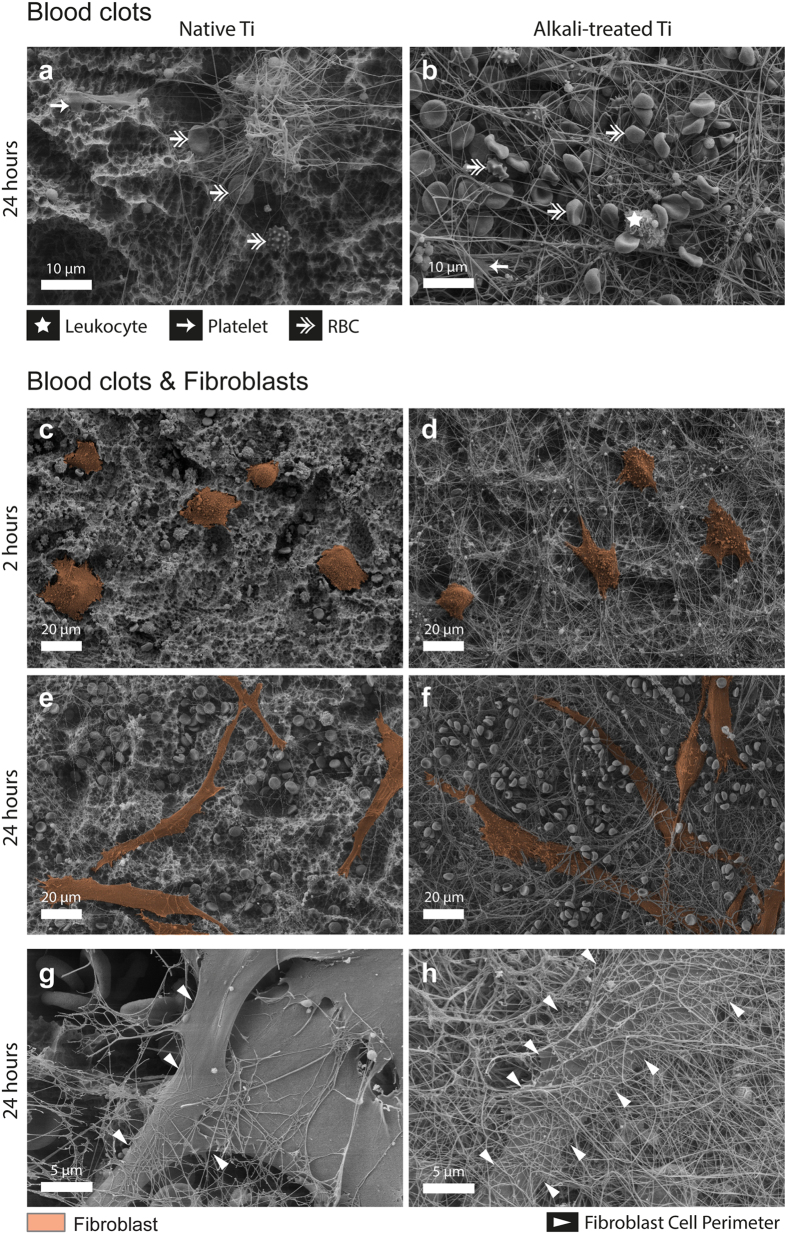
Alkali-treatment of Ti enhances blood clot formation and fibroblast integration into the surface-adhering blood clot. Scanning electron micrographs show the morphology of surface-adhering blood clots and of fibroblasts. (**a,b**) Blood clot morphologies after blood exposure for 2 hours, followed by 24 hours of further cultivation on (**a**) native and (**b**) alkali-treated Ti. Alkali-treatment enhances blood cell adhesion and fibrin matrix formation compared to native Ti surfaces. (**c–h**) Morphology of fibroblasts interacting with blood clots on native or alkali-treated Ti. False-coloured fibroblasts (brown) 2 h after seeding are spreading (**c,d)** and are fully spread and interacting with the blood clot 24 h after seeding (**e,f**). Higher magnification images of fully spread fibroblasts after 24 h (**g,h**) show the fibrillar extracellular matrix surrounding fibroblasts. Arrowheads point to the locations of fibroblast cell perimeters. Alkali-treatment of Ti was observed to increase blood clot formation and integration of fibroblasts into the blood clot ECM (**f,h**).

**Figure 2:**
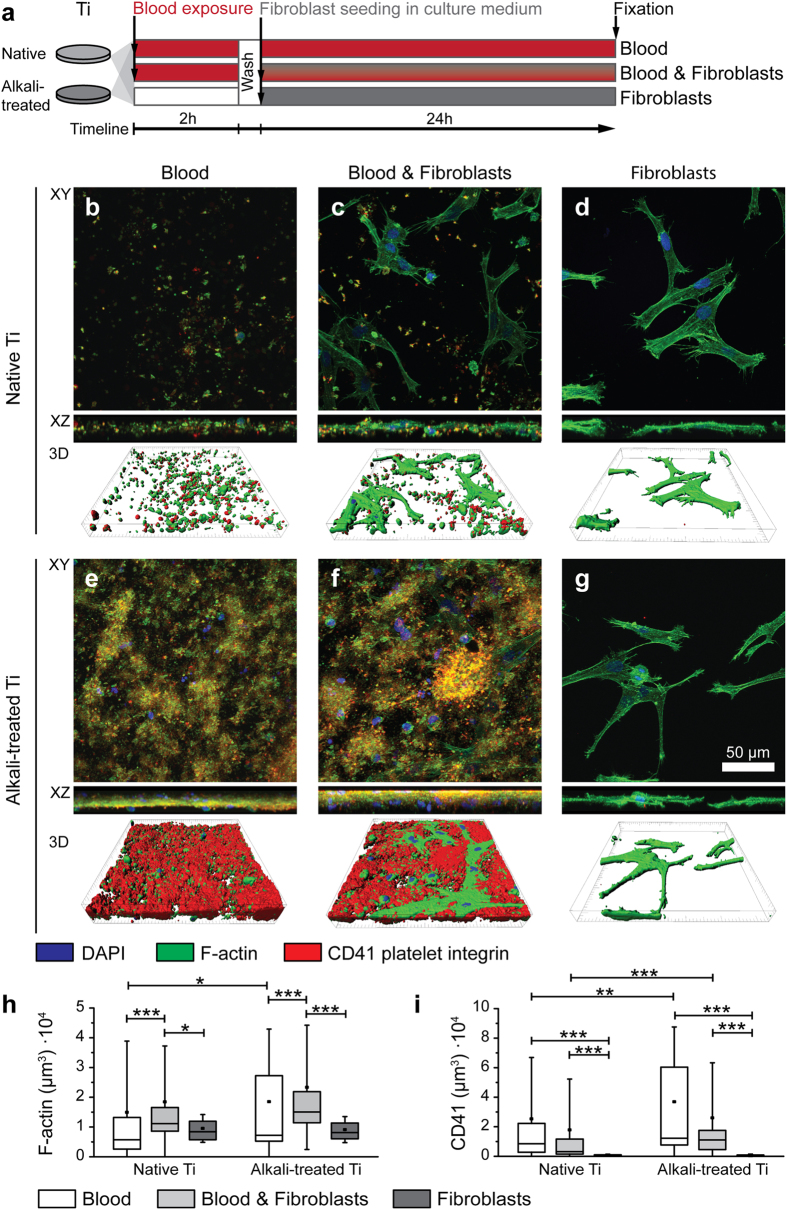
Alkali-treatment of Ti increases adhesion of blood cells. (**a**) Schematic drawing depicting the experimental procedure, timeline and naming of the experimental conditions. (**b–g)** Immunofluorescent micrographs of native (**b–d**) and alkali-treated (**e–g**) Ti surfaces, either exposed to blood (**b,e**), blood and subsequently seeded fibroblasts (**c,f**) or fibroblasts only (**d,g**), shown here 24 h after seeding fibroblasts. Samples were stained for cell nuclei (DAPI, blue), F-actin (green) and platelet integrin αIIb (CD41, red). One field of view of each condition (using the same magnification) from donor 4 is presented as XY (top) and XZ side view maximum intensity projections (middle) and as surface rendered 3D representations (bottom). Alkali-treatment of Ti was observed to increase platelet and leukocyte adhesion to the surfaces, resulting in formation of a more homogenous and dense blood clot with increased thickness (as shown in XZ side views) compared to native Ti. (**h,i**) Quantification of cellular components on native and alkali-treated Ti exposed to blood (white bars), blood & fibroblasts (grey bars) or fibroblasts (dark grey bars), 24 h after fibroblast seeding. Total cell volumes per field of view were quantified using F-actin staining (**h**). Platelet-specific cell volume contribution was estimated from CD41, platelet integrin αIIb stained volume (**i**). Reported values in boxplots represent mean (middle square), median (central line), 25^th^ to 75^th^ percentile (box) and standard deviation (whisker) of 7 fields of view of duplicate Ti surfaces per condition repeated for 5 donors. Statistically significant differences between conditions are indicated by (*) for p < 0.05, by (**) for p < 0.01 and by (***) for p < 0.001. Significantly increased amount of cellular components, as well as platelets were found on alkali-treated Ti compared to native Ti when exposed to blood.

Alkali-treatment of rough Ti surfaces induced the formation of a thicker blood clot, which contained higher densities of fibrous structures and entrapped blood cells compared to native Ti surfaces (Fig. 1a,b), a finding that is in agreement with previous observations^31^. Blood clots are not only composed of a fibrin matrix, but also include blood-borne cells, such as platelets, leukocytes and erythrocytes that get entrapped into the blood clot as it forms. As blood cells are major players during the early wound healing by secreting growth factors and cytokines, the amount of adhering platelets, leukocytes and fibroblasts was characterized for all conditions on native and alkali-treated Ti surfaces using immunostaining for F-actin, platelet-specific αIIb integrin (CD41) and nuclear counterstain DAPI (Fig. 2). An overview of the experimental procedure, the timeline and the different conditions are presented in a schematic (Fig. 2a). Maximum intensity projections of confocal z-stacks of native (Fig. 2b–d) and alkali-treated Ti surfaces (Fig. 2e–g) show that alkali-treatment, compared to native Ti, increases the amount of adhering platelets and leukocytes for conditions including blood (Fig. 2b,c,e,f). Furthermore, XZ side views highlight differences in clot thickness, i.e. native Ti surfaces are covered with single cells or a thin cell layer, whereas alkali-treated Ti generally shows an increased thickness of the aggregated cell layer (Fig. 2b–g). Neither co-culture with blood nor alkali-treatment of Ti appears to affect fibroblast morphology after 24 hours, when fibroblasts are fully spread (Fig. 2c,d,f,g).

Since alkali-treatment increased blood clot thickness and the number of entrapped blood-borne cells^31^ (Fig. 2), a quantitative comparison of cell types and cell numbers was performed. Using blood from 5 different donors, immunofluorescent stains of F-actin (Fig. 2h) and CD41 (platelet specific cell contribution) (Fig. 2i), were analysed based on volume quantifications. For native and alkali-treated Ti, the amount of F-actin (platelets, leukocytes and fibroblasts) was significantly higher in co-culture than in conditions of blood (p < 0.0001 (native), p = 0.0004 (alkali-treated)), or fibroblasts alone (p = 0.033 (native), p < 0.0001 (alkali-treated)). Additionally, significantly higher amounts of F-actin were detected for alkali-treated compared to native Ti for the blood only conditions (p = 0.023) (Fig. 2h). Platelets (CD41 volume) were found in significantly higher amounts for conditions with blood (p = 0.008), as well as with blood & fibroblasts (p = 0.0006) on alkali-treated Ti compared to native Ti (Fig. 2i).

### Enhanced blood clotting promoted fibroblast spreading and integration into blood clots

Two hours after fibroblast seeding onto blood clots that had formed on both native and alkali-treated Ti, fibroblasts were in the process of spreading and their protrusions were already entangled with the fibrin matrix of the clot (Fig. 1c,d, fibroblasts false-coloured in brown). Images for all conditions are shown in Supplementary Fig. S1(2h)' and S2 (24h) after fibroblast seeding. Fibroblast spreading on blood-exposed alkali-treated surfaces appeared to be guided by blood-borne fibrous matrix structures, such as the dendritic fibroblast morphology, which aligned with the fibres (Fig. 1d).

After prolonged fibroblast culture (24 hours), spindle-shaped fibroblasts were observed on native and on alkali-treated Ti (Fig. 1e–h). Compared to native Ti, alkali-treatment resulted in more fibroblasts to be partly covered by a densely packed fibrous network (Fig. 1g,h). Thick surface-adhering blood clots, as formed on alkali-treated Ti surfaces, promoted an enhanced fibroblast integration into the fibrous clot matrix, similar as observed for human bone cells^23^.

### Co-culture of fibroblasts with entrapped blood cells enhanced fibroblast proliferation

**Figure 3:**
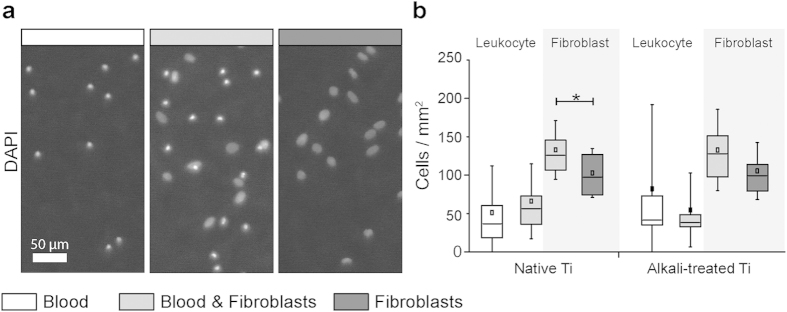
Co-culture of fibroblasts with blood clots enhances fibroblast proliferation. (**a)** Epi-fluorescence images of DAPI stained cell nuclei on native Ti surfaces for blood (white bar), blood & fibroblasts (grey bar) and fibroblasts (dark grey bar) conditions, 24 h after seeding fibroblasts. (**b**) Number of leukocytes and fibroblasts per mm^2^ counted from DAPI stained cell nuclei. Nuclei in blood & fibroblast co-culture conditions were differentiated for leukocyte and fibroblast nuclei according to nuclei size and intensity (leukocytes: smaller and brighter; fibroblasts: larger and dimmer). Reported values in boxplots represent mean (middle square), median (central line), 25^th^ to 75^th^ percentile (box) and standard deviation (whisker) of 11 images of 4 replicate surfaces per condition repeated for 5 blood donors. Statistically significant differences between conditions are indicated by (*) for p < 0.05. Co-culture of fibroblasts with blood increased fibroblast number compared to fibroblasts only conditions, which indicates a stimulating effect for fibroblast proliferation through blood clot interaction.

To further differentiate between adhering cell types, we quantified leukocyte and fibroblast numbers per mm^2^ (Fig. 3). This quantification included the analysis of multiple fields of view of quadruplicate surfaces for all conditions repeated with 5 blood donors. Separation of leukocyte and fibroblast nuclei in blood & fibroblasts co-culture conditions was achieved with a k-Means clustering algorithm, grouping nuclei according to nuclear size and fluorescent intensity, i.e. leukocyte nuclei were found to be small and bright, whereas fibroblast nuclei appeared larger and with lower intensity. Figure 3a shows representative epi-fluorescence DAPI images of blood, blood & fibroblasts and the fibroblast only conditions used for nuclei counting and clustering. Fibroblast numbers were similar on native and alkali-treated Ti surfaces for identical conditions. Interestingly, an increase in fibroblast number was observed in blood & fibroblast co-culture conditions compared to fibroblasts only, significant for native Ti (p = 0.019), but due to slightly larger variations, not significant (p = 0.083) for alkali-treated Ti. As no rinsing or medium exchange was performed, which could otherwise have removed non-adhering fibroblasts, increased fibroblast numbers as seen here indicate increased proliferation. Alkali-treatment did not appear to affect the morphology of the adhering fibroblasts, 24 h after seeding in the presence or absence of blood (Supplementary Fig. S2).

Additionally, large variations in adhering leukocytes on native and alkali-treated Ti surfaces were observed. Still, a trend to higher numbers of adhering leukocytes on alkali-treated versus native Ti surfaces was found, mainly for blood only conditions. An explanation could be that in blood & fibroblast co-culture conditions, enhanced remodelling of the blood clots has resulted in decreased leukocyte numbers in the currently used setup.

### Alkali-treatment enhanced ECM deposition, while co-culture of fibroblasts with blood clots enhanced blood clot matrix remodelling

**Figure 4:**
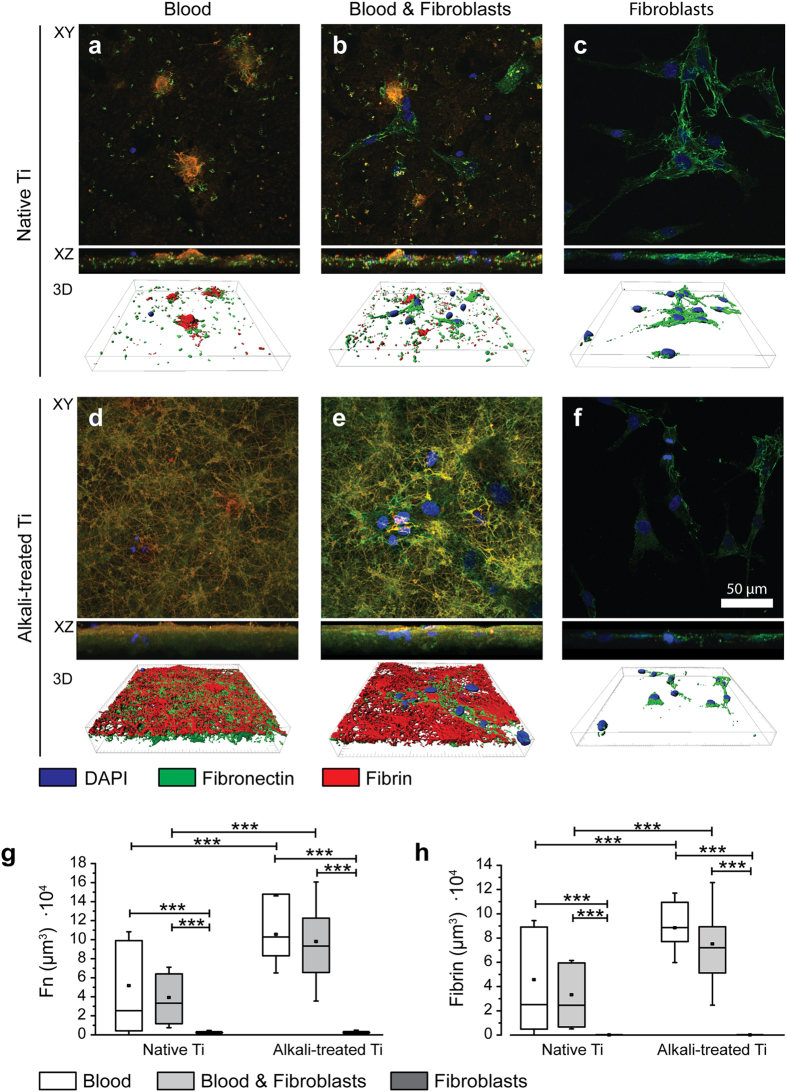
Alkali-treatment of Ti enhances ECM deposition, while co-culture of fibroblasts with blood clots induces ECM remodelling. (**a–f)** Immunofluorescent micrographs of native (**a–c**) and alkali-treated (**d–f**) Ti either exposed to blood (**a,d**), blood and subsequent seeded fibroblasts (**b,e**) or fibroblasts only (**c,f**), 24 h after seeding fibroblasts. Samples were immunostained for cell nuclei (DAPI) (blue), fibronectin (Fn) (green) and fibrin (red). One field of view of each condition (using the same magnification) from donor 4 is presented as XY (top) and XZ side view maximum intensity projections (middle) and as surface rendered 3D representations (bottom) at identical magnification. (**g,h**) Quantification of extracellular matrix components Fn **(g**) and fibrin (**h**) on native or alkali-treated Ti exposed to blood (white bars), blood & fibroblasts (grey bars) or fibroblasts (dark grey bars) 24 h after fibroblast seeding. Total ECM volumes per field of view were quantified from confocal micrograph z-stacks. Reported values in boxplots represent mean (middle square), median (central line), 25^th^ to 75^th^ percentile (box) and standard deviation (whisker) of 7 fields of view of duplicate Ti surfaces per condition repeated for 5 donors. Statistically significant differences between conditions are indicated by (***) for p < 0.001. Alkali-treatment of Ti significantly increased surface-adhering Fn and fibrin ECM compared to native Ti. Fibroblasts were shown to assemble new Fn matrix, either cultured on bare Ti or on blood clots. Remodelling of blood clots through fibroblasts was observed by trends in reduction of Fn and fibrin compared to blood only and fibroblasts residing in pits within blood clots. For timeline see Fig. 2a.

The stimulating effect of blood on fibroblast proliferation raises the question whether the presence of blood clots affects ECM amount and composition, and whether alkali-treatment of Ti contributs to this effect. Therefore, Fn, fibrin and cell nuclei were stained and the amount of Fn and fibrin was further quantified from confocal images (Fig. 4). Native Ti (Fig. 4a,b) of blood, as well as of blood & fibroblast co-culture conditions showed mainly separated small regions of Fn-fibrin fibrous network structures or non-fibrillar adsorbed protein layers compared to alkali-treated Ti surfaces (Fig. 4d,e), which were covered with dense, interconnected fibrous matrix, positive for highly overlapping fibrin and Fn.

**Figure 5:**
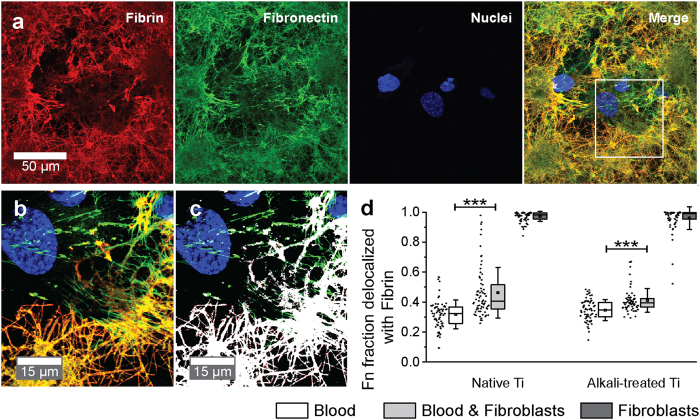
The blood clot matrix is composed of fibrin fibres, which colocalize to fibronectin, whereas newly assembled matrix is made of fibronectin only. (**a**) Immunofluorescent micrograph of blood and subsequent seeded fibroblasts on alkali-treated Ti surface represented as maximum intensity projections of a confocal z-stack (original 9.8 μm z-thickness) shown separate for fibrin, Fn, nuclei (DAPI) and as merged image. (**b**) Higher magnification image of a region of interest specified in (**a**) (merged image, white rectangle) showing merged maximum intensity projection of a substack of the original z-stack of 2 μm thickness. (**c**) Maximum intensity projection image as in (**b**) with Fn-Fibrin colocalized voxels depicted in white. (**d**) Quantification of Fn fraction delocalized with fibrin, which represents the remaining green voxels depicted in (**c**). Calculated fractions are shown as datapoints (left) and boxplots (right) for all conditions. Boxplots represent mean (middle square), median (central line), 25^th^ to 75^th^ percentile (box) and standard deviation (whisker) of 7 fields of view of duplicate Ti surfaces per condition repeated for 5 donors. Statistically significant differences between blood and blood & fibroblasts conditions are indicated by (***) for p < 0.001. Fibroblasts were shown to produce new Fn ECM and therefore significantly increase Fn fibrils delocalized with fibrin.

Quantification of Fn (Fig. 4g) and fibrin (Fig. 4h) volumes from confocal images revealed that most Fn and all fibrin originate from blood clots, whereas independent of surface treatment in fibroblasts only conditions, small amounts of newly synthesized and assembled Fn matrix were detected. Alkali-treatment of Ti caused a statistically significant increase in the amount of Fn and fibrin for blood containing conditions (all p < 0.0001). A decreasing trend of Fn and fibrin volumes (Fig. 4g,h), together with voids in co-culture compared to blood only conditions (Fig. 5a), suggest that fibroblasts actively degrade, remodel and integrate into dense blood clots. Newly assembled ECM (positive for Fn and delocalized with fibrin or fibrinogen) was found in the vicinity of fibroblasts (Fig. 5a, and higher magnification Fig. 5b,c). A quantification of these Fn fibres that did not colocalize with fibrin (Fig. 5d) showed statistically significant increases between blood only and blood & fibroblast conditions, for both Ti surface conditions (p < 0.0001 (native), p = 0.0031 (alkali-treated)). Alkali-treatment therefore contributes to the amount of fibrin and Fn deposited on Ti, primarily by increased blood coagulation. Already within 24 h, fibroblasts start to remodel the blood clot and synthesize new Fn matrix.

### Co-culture of blood & fibroblasts synergistically increased secreted MMPs and VEGF

**Figure 6:**
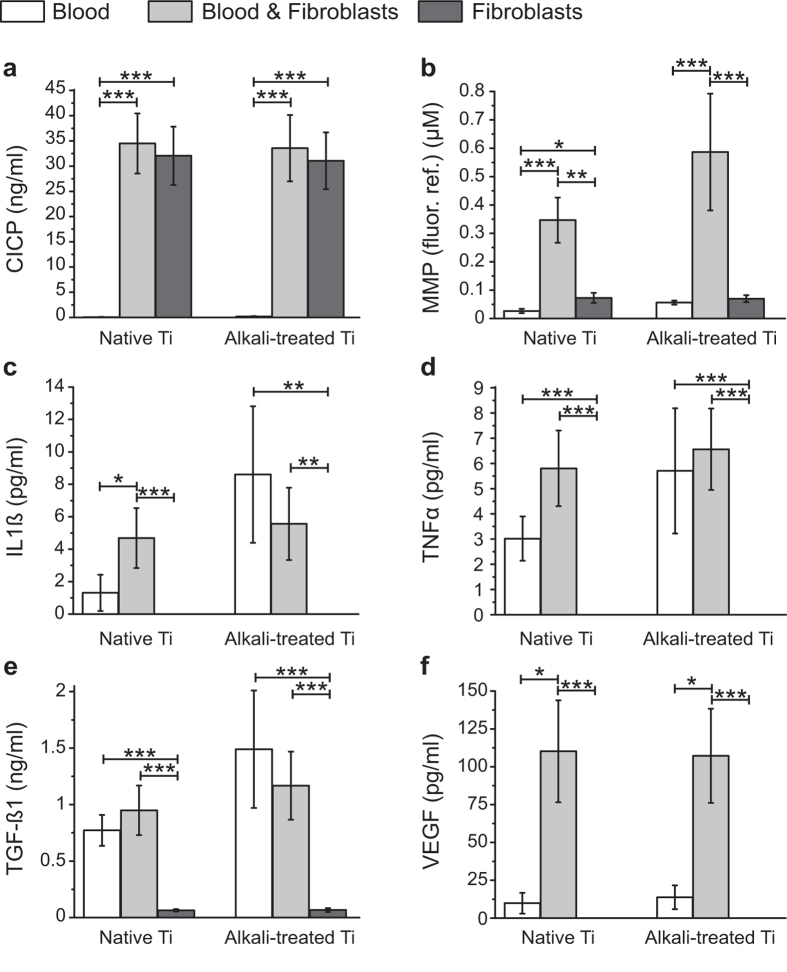
Co-culture of blood and fibroblasts synergistically increases secreted MMPs and VEGF. Concentration of soluble factors in the supernatants of native and alkali-treated Ti surfaces exposed to blood (white bars), blood & fibroblasts (grey bars) or fibroblasts (dark grey bars) 24 h after fibroblast seeding were quantified. Barplots show concentrations of (**a**) C-terminal peptide of pro-collagen type I (CICP), (**b**) total Matrix-metalloproteinase (MMP) including pro-MMP amount standardized to fluorescence reference substrate conversion, (**c**) IL1β, (**d**) TNFα, (**e**) TGF-β1 (including latent form) and (**f**) VEGF. Reported values correspond to mean values ± standard error of the mean for experiments repeated for 8 blood donors. Statistically significant differences between conditions are indicated by (*) for p < 0.05, by (**) for p < 0.01 and by (***) for p < 0.001. Collagen production represented by CICP concentrations originates from fibroblasts, i.e. values are similar between fibroblasts only and blood & fibroblast co-cultures. Alkali-treatment of Ti tends to increase concentrations of IL1β, TNFα, TGF-β1 and MMPs, although co-culture with fibroblasts tamed surface treatment differences of IL1β, TNFα and TGF-β1 concentrations. MMP and VEGF concentrations in fibroblast and blood co-culture conditions were significantly increased compared to blood or fibroblasts alone, suggesting a strong synergistic effect of fibroblasts and blood.

To obtain further support for the fast remodelling potential of fibroblasts, and to assess whether alkali-treatment had a beneficial effect on ECM production and remodelling, early collagen formation and matrix degrading proteases were investigated. Collagen production was assessed by quantification of the soluble C-terminal peptide of pro-collagen type I (CICP), which gets cleaved and released into the supernatant during collagen triple helix assembly. Matrix degrading proteases were detected with a generic MMP-assay. Alkali-treatment of Ti did not affect CICP levels, which was detected only when fibroblasts were present (Fig. 6a). Although, alkali-treatment of Ti compared to native Ti did not significantly increase MMP levels (only a trend was visible for blood as well as blood & fibroblast conditions, Fig. 6b), the co-culture of blood with fibroblasts had a significant impact on the amount of MMPs secreted, independent of Ti surface treatment (blood vs. blood & fibroblasts p < 0.0001 (native), p < 0.0001 (alkali-treated); fibroblasts vs. blood & fibroblasts p = 0.0016 (native), p < 0.0001 (alkali-treated)) (Fig. 6b). Furthermore, a set of important inflammatory cytokines and growth factors for wound healing was analysed by measuring soluble concentrations of cytokines (IL-1β and TNFα) and growth factors (TGF-β1 and VEGF). Assessed cytokines and growth factors were not detected or remained at low concentrations for the fibroblast only conditions (Fig. 6c–f), indicating their blood-associated origin. Although alkali-treatment of Ti showed trends of increasing cytokine levels and of TGF-β1 secretion, compared to native Ti for blood only conditions, these trends were diminished in co-culture conditions with fibroblasts (Fig. 6c–e). In contrast, VEGF as a prominent pro-angiogenic growth factor was detected at significantly higher concentrations in blood & fibroblast co-cultures, at similar high levels for native (p = 0.028 (vs. blood), p = 0.0002 (vs. fibroblasts)) and alkali-treated Ti (p = 0.032 (vs. blood), p = 0.0001 (vs. fibroblasts)), suggesting a synergistic interaction between blood and fibroblasts. These results support the hypothesis that blood clots highly support the regenerative functions of fibroblasts. Their synergy upregulates the secretion of MMPs and of VEGF.

### Donor-specific differences and similarities

To assess personalized differences, our experiments have been performed using blood from 8 different healthy donors (23–35 years old, 4 male and 4 female). The results showed relatively large standard deviations, which could be attributed to the large donor-to-donor variability not only for the observed experimental outcomes (see Supplementary Material for donor-specific immunofluorescence images of DAPI, F-actin, CD41 (Supplementary Fig. S3); DAPI, Fn, fibrin (Supplementary Fig. S4) and donor-specific quantifications of soluble factors (Supplementary Fig. S5)), but also for the initial screening parameters of the blood samples (Table 1). In addition, supernatants were pooled from 6 replicate Ti surfaces to obtain sufficient volume for quantification of soluble factors, and therefore replicate-specific differences were averaged out. All quantified data were analysed for statistical significant differences using 2-way ANOVA models with factor condition (native or alkali-treated Ti with either blood only, blood & fibroblasts or fibroblasts only) and factor donor, to account for donor-specific differences. Significant differences between donors were found for Fn, fibrin, CD41 and Fn fraction delocalized with fibrin, still showing highly significant differences between conditions.

## Discussion

**Figure 7:**
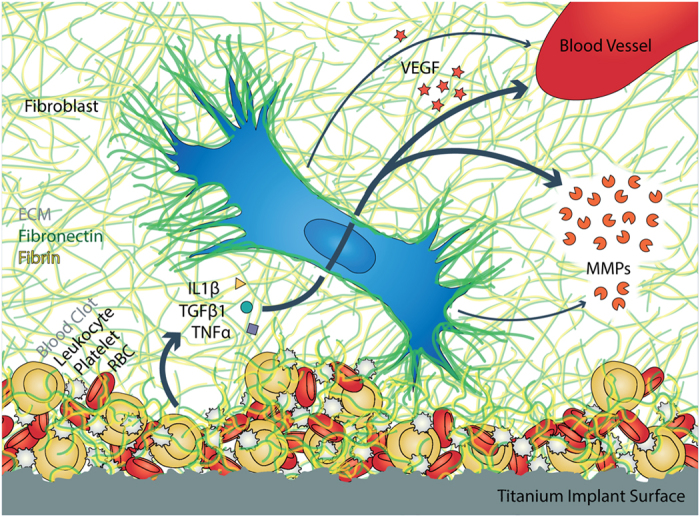
Suggested mechanism how fibroblasts and Ti surface-adhering blood clot synergistically upregulate remodelling capacity and angiogenic potential. A Ti implant surface provides a substrate for the blood clot that is composed of a fibrin-fibronectin ECM, various blood cells and a pool of growth factors (VEGF & TGF-β1), inflammatory cytokines (TNFα and IL1β) and matrix degrading enzymes (MMPs). Blood-associated MMP secretion accelerates the degradation of the blood clot, while secretion of the growth factor VEGF promotes angiogenesis. These data thus suggest a mechanism how the synergy between blood and invading fibroblasts contributes to the accelerated wound healing response on alkali-treated Ti surfaces.

Since little is known how initial surface-induced blood activation might promote tissue healing around implants, we asked whether the first contact of surfaces with blood might steer the later tissue response to materials. Our results support the hypothesis that the presence of a blood clot can indeed direct further wound healing processes as sketched in Fig. 7: First, the composition and morphology of the clot is dependent on material surface properties (Figs 1 and 2). Alkali-treatment of Ti surfaces, which renders surfaces from hydrophobic to superhydrophilic, increased the deposition of fibrin matrix leading to thicker blood clots compared to native Ti (Fig. 4), in agreement with previous observations^23^,^31^. Alkali-treatment increases the activation of the blood coagulation cascade directly via the intrinsic contact activation pathway, thus leading to increased fibrin deposition^31^. Second, the number of adhering platelets is significantly increased on alkali-treated compared to native Ti surfaces (Fig. 2b–g,i). This finding is particularly important in the context of the immune response, as well as of wound healing and tissue repair processes^12^,^15^,^32^, since platelets secrete a plethora of factors upon activation, including PDGF, TGFβ and VEGF^10^,^12^. Third, Fn was found to colocalize with the fibrillar fibrin clot matrix in early stages (Figs 4 and 5). Covalent cross-linking of plasma Fn and fibrin through the enzymatic activity of activated transglutaminase FXIII^33^, which in turn is activated by the coagulation cascade^34^, might further stabilise Fn-fibrin interactions, thereby promoting fibroblast adhesion, spreading and migration^34^,^35^,^36^. At later stages, fibroblasts were observed to assemble new Fn fibres in the presence as well as in the absence of blood clots (Figs 4 and 5). Fourth, co-culture of fibroblasts with entrapped blood cells enhanced fibroblast proliferation (Fig. 3). Fifth, the synergies between blood clot with entrapped blood cells and fibroblasts significantly upregulated the secretion of MMPs (Fig. 6b) and therefore the degradation of the clot matrix. Sixth, the blood clots with entrapped blood cells serve as a source of growth factors and cytokines, i.e. of VEGF, TGF-β1, IL1β and TNFα (Fig. 6c–f). Whereby the highest levels in the overall expression of VEGF were detected when the whole blood clots synergised with invading fibroblasts (Fig. 6f).

Contrary to platelets, leukocyte numbers were similar on native and alkali-treated Ti surfaces after 24 hours (Fig. 3b). However, using the same setup, alkali-treatment of Ti was previously shown to significantly increase leukocyte numbers (presumably neutrophils) in adhering blood clots after 2 h blood exposure^31^. Hence, the additional 24 hours culture time in the current work may have resulted in neutrophil clearance by phagocytic macrophages^13^,^14^. The importance of neutrophils in wound healing was demonstrated *in vitro,* i.e. neutrophils from wound sites regulate the innate immune response by modulating phenotype and cytokine profile expression of macrophages^16^. Additionally, wounds of neutrophil-depleted mice showed delayed wound closure, presumably caused by a lack of stimuli from apoptotic neutrophils to activate macrophages^37^. Even though apoptosis is a frequently noted event during normal wound healing, specific analysis of apoptosis was not performed in this study, and hence, an enhanced induction of apoptosis through interaction of the blood cells and fibroblasts with the Ti surface can not be excluded.

Blood clots with entrapped blood cells act as a major source of growth factors and of pro-inflammatory cytokines, i.e. TGF-β1, IL1β and TNFα (Fig. 6c–e). Apart from IL1β on native Ti surfaces, levels of TGF-β1, IL1β and TNFα were comparable for blood only conditions and the blood clots seeded with fibroblasts. The detected levels very likely originate from activated platelets, which are known for rapidly inducing and sustaining the synthesis of IL-1β over several hours^38^, and from activated macrophages that are known to release pro-inflammatory cytokines such as TNFα, IL1β, IL12^39^ and growth factors (e.g. TGF-β, basic fibroblast growth factor (bFGF), PDGF)^15^. TGF-β1 is known to mediate tissue repair and wound healing as an anti-inflammatory cytokine^15^, in addition to its effect on fibroblast differentiation into myofibroblasts^40^ required for wound contraction. In the current study, TGF-β1 concentrations were measured in the range of 0.75–1.5 ng/ml, which is comparable to described concentrations (2–10 ng/ml) inducing fibroblast to myofibroblast differentiation over the time course of 3 or more days^40^. However, immunostaining did not show any expression of α-SMA by fibroblasts, a marker for myofibroblast differentiation^41^, at least not after 24 hours.

Major crosstalk between entrapped blood cells and invading fibroblasts resulted in an increased remodelling capacity through enhanced secretion of MMPs. Although MMP expression was detected for blood only as well as fibroblast only conditions (Fig. 6b), as expected^42^,^43^, it was highly upregulated in the co-culture of blood & fibroblasts (Fig. 6). MMPs enable functional remodelling of provisional matrix during normal wound healing and inhibition was shown to delay wound contraction, myofibroblast differentiation, as well as blood vessel formation^44^. MMPs in the blood only condition among others originate from neutrophils, which secrete neutrophil elastase and MMP-9 upon activation^45^. In addition, MMP secretion by monocytes and macrophages is upregulated after adhesion to preformed ECM, or ECM components such as Fn, collagen or laminin, and is further enhanced upon binding to platelets^46^. Although fibroblasts are known to synthesize MMPs, exposure to Fn was also shown to further enhance MMP secretion^43^, which might explain the synergistic increase in detected MMPs. Furthermore, cytokines or growth factors that originate from the blood clot (possibly but not limited to TGF-β1, IL1β or TNFα) may have further triggered this synergistic response. Particularly, proinflammatory cytokines, as secreted by neutrophils, monocytes/macrophages and platelets, induce MMP expression, especially in wound sites^15^. Our data therefore suggest that MMP secretion is increased by fibroblasts in response to pro-inflammatory cytokines and their interaction with the blood clot matrix.

Our most striking observation is that the synergy between blood clots and invading fibroblasts gives rise to significantly increased levels of VEGF (Fig. 6f). Angiogenesis is crucial for wound healing^47^, and the development of methods to increase angiogenesis in the proximity of implants remains one of the most challenging problems in tissue engineering and regenerative medicine^48^. One common approach with limited clinical success was thus to furbish implants with bioactive coatings^49^. The synergistic upregulation of VEGF is therefore highly significant, since it can explain the poor predictive clinical outcome of cell monoculture experiments. VEGF is released from polymorphonuclear cells (PMNs, such as neutrophils) and macrophages^15^ during the early tissue maturation phase^50^, and mediates the recruitment of endothelial progenitor cells, endothelial cell proliferation, survival, migration and differentiation^51^,^52^. Additionally, platelets contain VEGF in their alpha granules together with other angiogenic factors, which collectively stimulate fibroblast and endothelial cell proliferation^12^. A recent study, investigating the interaction of peripheral blood mononuclear cells (PBMCs) with mesenchymal stem cells (MSCs) seeded onto scaffolds, also found a synergistic upregulation of VEGF upon PBMC-MSC co-culture compared to VEGF secreted by either cell type alone, even though MSCs alone secreted substantial amounts^53^. In the current study, upregulation of VEGF was only detected when blood was co-cultured with fibroblasts (Fig. 6f).

These same synergies between blood and invading fibroblasts thus provide a mechanistic explanation why alkali-treatment of Ti promotes healing of alkali-treated dental implants (Fig. 7), especially of endosseous healing as clinically observed^25^,^26^,^29^,^30^: First, alkali-treatment increases the blood clot thickness and thus the density of entrapped blood cells, including neutrophils (Fig. 2)^31^. This in turn accelerates the invasion of fibroblasts which subsequently upregulates the expression of MMPs (Fig. 6b) that are crucial for the degradation of the provisional clot matrix and for the assembly of new ECM. The synergistic interactions of blood-borne cells and fibroblasts, as well as of the stimulatory roles of peptide fragments released from the degrading blood clot subsequently upregulate VEGF release (Fig. 6f) and thus the angiogenic potential. Finally, angiogenesis plays a major role in bone repair^54^,^55^. In contrast and in the absence of a blood clot, no differences were seen in the behaviour of fibroblasts on alkali-treated versus native Ti surfaces (Fig. 2). This is in agreement with the literature, where osteoblastic cells (human bone marrow stromal cells, MG-63 and SaOS-2) showed no morphological differences on native versus alkali-treated Ti *in vitro*^56^, and human gingival fibroblasts showed increased adhesion on alkali-treated Ti after 1 h, however these differences diminished after 3 hours *in vitro*^57^.

For future work and when focusing on osseointegration of Ti implants, this method of including initial blood-material interactions should further be applied to osteoblasts and/or stem cells, which would require more extended time periods to allow for cell differentiation. This agrees with a study from Bae *et al.*, who showed that physical cues from the ECM can stimulate differentiation of preosteoblasts and MSCs^58^. In addition, the use of blood from patients who suffer from a high prevalence of implant failure (i.e. diabetic patients), may provide additional insights into the underlying mechanisms, and assist in improving implant design.

The specific findings made here on Ti surfaces might be of far broader relevance. Since most implants get into contact with blood during surgery, negligence of the healing potential from the crosstalk between blood-borne cells, initial provisional fibrin-Fn matrix, secreted growth factors and cytokines and the first generation of invading cells might be the key reason why conventional cell culture systems, typically conducted with one or two cell types only, have limited clinical predictive power regarding the performance of biomaterials and their physicochemical surface properties. This study design of co-culturing cells of interest together with blood clots is shown here to improve on the predictive power regarding the healing potential of biomaterials. We thus recommend that this approach is implemented to test new materials designed for implantation, before moving towards *in vivo* testing and reducing animal experiments that will fail because of wrong predictions *in vitro*.

## Methods

### Preparation of titanium surfaces

Disk-shaped Ti surfaces of 15 mm in diameter and 2 mm thickness composed of titanium grade 4 (Dynamet Incorporated Washington) were prepared, sandblasted, acid-etched (SBA) by Thommen Medical AG and used as native Ti or further alkali-treated as previously described^31^,^59^. Previous surface analysis indicated that superhydrophilicity created by alkali-treatment of microrough SBA Ti surfaces might partly be attributed to deprotonation and ion exchange of hydroxyl-groups on the TiO_2-x_ surfaces increasing the ionic character and the net negative surface charge^59^. In brief, alkali-treatment was performed by sonication of Ti surfaces in 0.05 M aqueous NaOH for 30 s at room temperature immediately before the experiment.

### Blood collection and screening analysis

Study approval from the local Ethical Committee of the Kanton Zurich (KEK Zurich) was received under No. 2012-0111. The methods were carried out in accordance with the approved guidelines. Whole blood from healthy volunteers was obtained at the University Hospital Zurich after receiving informed consent according to standardized guidelines. For experimental reasons, blood samples were anti-coagulated with heparin at a low concentration. Up to 60 ml of venous blood per donor was collected in vacutainer tubes (BD Vacutainer™ No Additive (Z) Plus Tubes) and heparinized upon withdrawal to a final concentration of 3IU sodium heparin (Carl Roth, Germany, Eur. Ph., 120 IU/mg) per ml blood. Blood of every donor was screened for hematogram with automated leukocyte differentiation, coagulation status and fibrinogen concentration by the Institute for Haematology and for Haemoglobin A1c (HbA1c) by the Institute for Clinical Chemistry of the University Hospital Zurich, as a measure for non-diagnosed diabetic conditions. The volunteers were classified as healthy as analysed parameters were within the normal clinical range.

### Blood clot formation

To mimic most closely a surgical situation, where an implant material gets into contact with the patient’s blood, we used fresh human whole blood within 2 hours after it had been drawn. Native and alkali-treated SBA Ti surfaces were incubated with whole blood in a rotating slide chamber system, as previously described^31^. In brief, Ti discs were assembled on both sides of a Teflon ring (inner diameter 12 mm, height 10 mm) and clamped between two stainless steel plates creating a circular chamber with a final volume of 1.1 ml. The closed chambers were filled completely with blood through a syringe port and incubated for 2 hours under rotation at 6.6 rpm in an incubator (B6030, Heraeus, Hanau, Germany) at 37 °C. The surfaces were rinsed 3 times in PBS with Ca^2+^ (0.9 mM CaCl) and Mg^2+^ (0.49 mM MgCl) and subsequently used for cell culture experiments.

### Culture of human fibroblasts

Normal human dermal fibroblasts from juvenile foreskin (NHDF-c, C-12300, PromoCell, Heidelberg, Germany) were cultured in Modified Eagle Medium Alpha (MEMalpha, No. L0475, Biowest) supplemented with 2% (v/v) Human serum (converted) type AB (No. C11-002, Lot No. C00209-2663, PAA, Pasching, Austria) without iso-agglutinins to eliminate blood type dependent immune responses. Cells were cultured at 37 °C and 5% CO_2_ in a humidified atmosphere. The medium was changed every two to three days. Cells of subculture passage number 8 were used for all experiments. Near-confluent fibroblasts were released from TCP flasks (TPP) with Accutase (A6964, Sigma), centrifuged, resuspended in culture medium and seeded onto bare or blood-exposed Ti surfaces in 24-well plates at a seeding density of 7500 cells/cm^2^. Blood only, blood with subsequently seeded fibroblasts and fibroblasts only on native and alkali-treated Ti surfaces were maintained in culture for 2 or 24 hours.

### Scanning electron microscopy

SBA Ti surfaces, 2 or 24 hours after fibroblast seeding, were analysed by scanning electron microscopy. Briefly, following blood incubation and/or subsequent fibroblast culture, Ti surfaces were rinsed once with PBS (without Ca^2+^ and Mg^2+^), fixed with glutaraldehyde (4% (v/v) in PBS) for 1 h and rinsed 3 times with PBS. Further fixation with osmium tetroxid (OsO_4_, 0.5% (v/v) in ddH_2_O) for 1 h was followed by dehydration in a graded series of ethanol (from 50% to 100% in ddH_2_O). Subsequently, Ti surfaces were dried over the critical point of CO_2_ (Tc: 31 °C, Pc: 73.8 bar) using a critical-point dryer (CPD 030, Bal-Tec AG, Balzers, Liechtenstein or Tousimis CDP 931, Rockville, USA). After sputtercoating with 5 nm platinum (MED010, Balzers) images were recorded with a Zeiss Leo-1530 scanning electron microscope at 5 kV acceleration voltage detecting secondary electron signals.

### Immunofluorescent staining and fluorescence microscopy

After culturing of blood, blood & fibroblasts or fibroblasts on native and alkali-treated Ti surfaces for 2 or 24 h, Ti surfaces were rinsed once with PBS and subsequently fixed in 4% (v/v) paraformaldehyde in PBS for 20 min followed by three rinses with PBS. Only for F-actin and CD41 staining, cells were permeabilized with 0.1% (v/v) Triton-X 100 with 0.5% (w/v) bovine serum albumin (BSA) in PBS for 10 min and followed (for all stainings) by blocking with 2% (w/v) BSA in PBS for 1 h. Used antibodies were incubated at room temperature and diluted in 2% (w/v) BSA in PBS. For F-actin and CD41 staining, 1:100 diluted CD41 mouse monoclonal anti-integrin α2b antibody (I9660, Sigma Aldrich) was incubated for 1 h, followed by 2 rinses with 2% (w/v) BSA in PBS and 2 rinses with PBS. Subsequently 1:200 diluted goat anti-mouse IgG Alexa 633 (Molecular Probes) and Alexa Fluor 488 phalloidin (A12379, Invitrogen) were incubated for 1 h. For staining of Fn and fibrin, 1:100 diluted sheep anti-human fibronectin (AHP08, AbD serotec) and mouse monoclonal anti-human fibrin(ogen) (F9902, Sigma) antibodies were incubated for 1 h prior to rinsing as described before. Incubation of secondary antibodies donkey anti-sheep IgG Alexa 488 (1:200, Molecular Probes) and goat anti-mouse IgG Alexa 555 (1:200, Molecular Probes) for 1 h followed. Subsequently Ti surfaces were washed 3 times with PBS, stained for cell nuclei with 5 μg/ml 4′,6-diamidino-2-phenylindole, dilactate (DAPI, D3571, Invitrogen) in PBS for 5 min and rinsed with PBS. Ti surfaces were mounted on glass coverslips (24 × 50 mm, Carl Roth) using Mowiol 4–88 mounting media (Calbiochem) and analysed with an Olympus FV1000 laser scanning confocal microscope using a 60 × 1.35 NA oil immersion objective with a field of view of 44931.1 μm^2^. Lower magnification images for cell nuclei quantification were acquired with a Nikon TE2000-E epi-fluorescence microscope using a 10 × 0.3 NA air objective.

### Image analysis and quantification

Confocal z-stack images of cell (F-actin and CD41) and ECM (Fn and fibrin) components on Ti surfaces were quantified using Fiji^60^. Briefly, separate channels of z-stacks have been thresholded using stack histogram based Otsu’s method, with a fixed lower limit of 300. Voxels above the threshold were taken as positive for the respective channel, quantified and converted to volume. Colocalized voxels of fibrin and Fn were quantified on logical AND combined masks of the two separate channels. Analyses included 7 stacks of different fields of view per surface for duplicate Ti surfaces per condition repeated for 5 different blood donors, resulting in n = 70 analysed stacks per condition. 3D representations of z-stacks were created by surface rendering confocal data with Imaris ×64, 7.5.1, software (Bitplane AG, Zurich, Switzerland) based on intensity thresholds. Number of leukocytes and fibroblasts were analysed from low-magnification images of DAPI stained nuclei. Masks of cell nuclei were created after background subtraction, applying local threshold (Bernsen method) and analysis of particles with size of 12–2000 pixels and circularity of 0.1–1.0. Created masks were applied to original images to get measures of total nuclei number as well as size and grey level intensity for single nuclei. For images from blood & fibroblast conditions, measured area, mean grey and maximum intensity values of all nuclei were used as input parameters for a k-Means clustering algorithm applied in MATLAB R2013b (The MathWorks, Inc., Natick, Massachusetts, USA) resulting in separation of fibroblast and leukocyte nuclei into 2 clusters. Fibroblast and leukocyte quantification represents 11 images (area = 0.64 mm^2^) for 4 Ti surfaces per condition repeated for 5 blood donors, resulting in n = 220 images per condition.

### Quantification of soluble factors in supernatant

Supernatants of 6 Ti surfaces per condition for every blood donor were collected 24 h after fibroblast seeding and pooled. Pooled supernatants were centrifuged at 1000 g for 15 minutes at 4 °C, subsequently aliquoted and stored at −80 °C until further analysis. Supernatants were analysed with Elisa assays for CICP pro-collagen type I (Quidel, MicroVue CICP EIA, 8003), total direct acid-activated TGF-β1 (Promega, TGF-β1 Emax ImmunoAssay System, G7590), IL-1β (BD OptEIA Human IL-1beta ELISA Set II, No. 557953), TNFα (BD OptEIA Human, No. 555212) and VEGF (Invitrogen, VEGF Human Antibody Pair, No. CHG0113, with Antibody Pair Buffer Kit (CNB0011)). MMPs including pro-MMPs activated for 2 hours before the assay were quantified using a fluorometric MMP generic assay (Anaspec, SensoLyte 520 generic MMP assay kit, No. 71158). All assays were performed according to the manufacturer’s instructions measuring supernatant samples in duplicates.

### Statistical analysis

All values were analysed for statistical significance using 2-way ANOVA models with factors donor and condition after checking Q-Q plots and Tukey-Anscombe distribution plots and log-transformation to ensure normality distribution of residuals (apart from Fig. 5d) using the programme R. Statistical differences between groups were tested with Tukey post-hoc comparison and statistical significance between conditions was accepted for *p* < 0.05 denoted with (*), p < 0.01 (**) and p < 0.001 (***).

## Additional Information

**How to cite this article**: Burkhardt, M. A. *et al.* Synergistic interactions of blood-borne immune cells, fibroblasts and extracellular matrix drive repair in an *in vitro* peri-implant wound healing model. *Sci. Rep.* **6**, 21071; doi: 10.1038/srep21071 (2016).

## Supplementary Material

Supplementary Information

## Figures and Tables

**Table 1:** Blood of all 8 donors was screened for hematogram with automated leukocyte differentiation, coagulation status and fibrinogen concentration, as well as concentration of Haemoglobin A1c (HbA1c) to exclude for non-diagnosed diabetic conditions.

Parameter	Unit	Normal range	Donor values								Mean	SD
Donor	Male (m)/Female (f)		1	2	3	4	5	6	7	8		
Gender			m	m	m	f	m	f	f	f		
Haemoglobin	g/l	117–170	161	133	144	120	157	123	116	136	136.3	16.7
Haematocrit	l/l	0.35–0.5	0.47	0.39	0.45	0.39	0.46	0.37	0.35	0.39	0.41	0.04
Erythrocytes	10^12^/l	3.9–5.7	5.3	4.46	6.72	5.26	5.19	4.94	5.97	4.64	5.31	0.73
Platelets	10^9^/l	143–400	305	222	252	279	172	233	394	264	265.1	65.6
Leukocytes	10^9^/l	3.0–9.6	5.79	3.62	8.16	5.42	5.08	5.18	7.85	5.46	5.82	1.50
Neutrophils	10^9^/l	1.4–8.0	2.94	1.67	4.1	3.13	3.11	2.83	3.88	3.5	3.15	0.75
Monocytes	10^9^/l	0.16–0.95	0.39	0.56	0.42	0.4	0.22	0.43	0.63	0.4	0.43	0.12
Eosinophils	10^9^/l	0.0–0.7	0.08	0.06	0.26	0.04	0.08	0.2	0.21	0.05	0.12	0.09
Basophils	10^9^/l	0.0–0.14	0.02	0.02	0.03	0.01	0.03	0.05	0.07	0.03	0.03	0.02
Lymphocytes	10^9^/l	1.5–4.0	2.36	1.31	3.35	1.84	1.57	1.67	3.06	1.48	2.08	0.76
Quick	%	>70	87	87	93	109	92	—	97	98	94.7	7.6
INR	—	<1.2	1.1	1.1	1.1	1	1.1	—	1.1	1.1	1.1	0.0
aPTT	s	24–36	24	27	28	24	25	—	23	25	25.1	1.8
Fibrinogen	g/l	1.5–4.0	2	2.8	2.2	3.8	2.4	—	3.3	2	2.6	0.7
HbA1c	%	4.8–5.9	5.1	5	5.8	5	5.3	5.4	4.7	5	5.2	0.3

Table 1 shows measured values for all 8 donors, as well as the mean values and standard deviations (SD) for all donors. Used blood donors were classified as healthy as analysed parameters were not considered pathological.

## References

[b1] Gurtner G. C., Werner S., Barrandon Y. & Longaker M. T. Wound repair and regeneration. Nature 453, 314–321 (2008). 18480812 10.1038/nature07039

[b2] Turbill P., Beugeling T. & Poot A. Proteins involved in the Vroman effect during exposure of human blood plasma to glass and polyethylene. Biomaterials 17, 1279–1287 (1996). 8805975

[b3] Schwarz F. *et al.* Potential of chemically modified hydrophilic surface characteristics to support tissue integration of titanium dental implants. J. Biomed. Mater. Res. Part B Appl. Biomater. 88, 544–557 (2009). 18837448 10.1002/jbm.b.31233

[b4] Gorbet M. & Sefton M. Biomaterial-associated thrombosis: roles of coagulation factors, complement, platelets and leukocytes. Biomaterials 25, 5681–5703 (2004). 15147815 10.1016/j.biomaterials.2004.01.023

[b5] Milleret V., Hefti T., Hall H., Vogel V. & Eberli D. Influence of the fiber diameter and surface roughness of electrospun vascular grafts on blood activation. Acta Biomater 8, 4349–4356 (2012). 22842036 10.1016/j.actbio.2012.07.032

[b6] Laurens N., Koolwijk P. & De Maat M. P. M. Fibrin structure and wound healing. J Thromb Haemost 4, 932–939 (2006). 16689737 10.1111/j.1538-7836.2006.01861.x

[b7] Cooper L. F. A role for surface topography in creating and maintaining bone at titanium endosseous implants. J Prosthet Dent 84, 522–534 (2000). 11105008 10.1067/mpr.2000.111966

[b8] Arwert E. N., Hoste E. & Watt F. M. Epithelial stem cells, wound healing and cancer. Nat Rev Cancer 12, 170–180 (2012). 22362215 10.1038/nrc3217

[b9] Chen W.-J. *et al.* Cancer-associated fibroblasts regulate the plasticity of lung cancer stemness via paracrine signalling. Nat Commun 5, 3472 (2014). 24668028 10.1038/ncomms4472

[b10] Nurden A. T., Nurden P., Sanchez M., Andia I. & Anitua E. Platelets and wound healing. Front Biosci 13, 3532–3548 (2008). 18508453 10.2741/2947

[b11] Zarbock A., Polanowska-Grabowska R. K. & Ley K. Platelet-neutrophil-interactions: linking hemostasis and inflammation. Blood Rev 21, 99–111 (2007). 16987572 10.1016/j.blre.2006.06.001

[b12] Blair P. & Flaumenhaft R. Platelet alpha-granules: basic biology and clinical correlates. Blood Rev 23, 177–189 (2009). 19450911 10.1016/j.blre.2009.04.001 PMC2720568

[b13] Bratton D. L. & Henson P. M. Neutrophil clearance: when the party is over, clean-up begins. Trends Immunol 32, 350–357 (2011). 21782511 10.1016/j.it.2011.04.009 PMC3151332

[b14] Meszaros A. J., Reichner J. S. & Albina J. E. Macrophage phagocytosis of wound neutrophils. J Leukocyte Biol 65, 35–42 (1999). 9886244 10.1002/jlb.65.1.35

[b15] Eming S. A., Krieg T. & Davidson J. M. Inflammation in wound repair: molecular and cellular mechanisms. J Invest Dermatol 127, 514–525 (2007). 17299434 10.1038/sj.jid.5700701

[b16] Daley J. M. *et al.* Modulation of macrophage phenotype by soluble product(s) released from neutrophils. J Immunol 174, 2265–2272 (2005). 15699161 10.4049/jimmunol.174.4.2265

[b17] Stanford C. M. Surface modification of biomedical and dental implants and the processes of inflammation, wound healing and bone formation. Int J Mol Sci 11, 354–369 (2010). 20162020 10.3390/ijms11010354 PMC2821008

[b18] Hong J. *et al.* Titanium is a highly thrombogenic biomaterial: possible implications for osteogenesis. Thromb. Haemost. 82, 58–64 (1999). 10456455

[b19] Kokubo T., Kim H. & Kawashita M. Novel bioactive materials with different mechanical properties. Biomaterials 24, 2161–2175 (2003). 12699652 10.1016/s0142-9612(03)00044-9

[b20] Anderson J. M. Biological Responses to Materials. Annu. Rev. Mater. Res. 31, 81–110 (2001).

[b21] Arvidsson S., Askendal A. & Tengvall P. Blood plasma contact activation on silicon, titanium and aluminium. Biomaterials 28, 1346–1354 (2007). 17156838 10.1016/j.biomaterials.2006.11.005

[b22] Gillitzer R. & Goebeler M. Chemokines in cutaneous wound healing. J Leukocyte Biol 69, 513–521 (2001). 11310836

[b23] Kopf B. S., Schipanski A., Rottmar M., Berner S. & Maniura-Weber K. Enhanced differentiation of human osteoblasts on Ti surfaces pre-treated with human whole blood. Acta Biomater 19, 180–190 (2015). 25818948 10.1016/j.actbio.2015.03.022

[b24] Shiu H. T., Goss B., Lutton C., Crawford R. & Xiao Y. Formation of blood clot on biomaterial implants influences bone healing. Tissue Eng Pt B-Rev 20, 697–712 (2014). 10.1089/ten.TEB.2013.0709 24906469

[b25] Stadlinger B. *et al.* Surface-conditioned dental implants: an animal study on bone formation. J. Clin. Periodontol. 36, 882–891 (2009). 19735467 10.1111/j.1600-051X.2009.01466.x

[b26] Stadlinger B. *et al.* Biomechanical evaluation of a titanium implant surface conditioned by a hydroxide ion solution. Br J Oral Maxillofac Surg 50, 74–79 (2012). 21177005 10.1016/j.bjoms.2010.11.013

[b27] Buser D. *et al.* Enhanced bone apposition to a chemically modified SLA titanium surface. J. Dent. Res. 83, 529–533 (2004). 15218041 10.1177/154405910408300704

[b28] Calvo-Guirado J. L. *et al.* Histological and histomorphometric evaluation of immediate implant placement on a dog model with a new implant surface treatment. Clin Oral Implants Res 21, 308–315 (2010). 20074244 10.1111/j.1600-0501.2009.01841.x

[b29] Vasak C. *et al.* Early bone apposition to hydrophilic and hydrophobic titanium implant surfaces: a histologic and histomorphometric study in minipigs. Clin Oral Implants Res doi: 10.1111/clr.12277 (2013). 24118429

[b30] Held U., Rohner D. & Rothamel D. Early loading of hydrophilic titanium implants inserted in low-mineralized (D3 and D4) bone: one year results of a prospective clinical trial. Head Face Med 9, 37 (2013). 24321192 10.1186/1746-160X-9-37 PMC3866303

[b31] Milleret V., Tugulu S., Schlottig F. & Hall H. Alkali treatment of microrough titanium surfaces affects macrophage/monocyte adhesion, platelet activation and architecture of blood clot formation. Eur Cell Mater 21, 430–444 (2011). 21604243 10.22203/ecm.v021a32

[b32] Herter J. M., Rossaint J. & Zarbock A. Platelets in inflammation and immunity. J Thromb Haemost 12, 1764–1775 (2014). 25224706 10.1111/jth.12730

[b33] Mosher D. F. Cross-linking of cold-insoluble globulin by fibrin-stabilizing factor. J Biol Chem 250, 6614–6621 (1975). 1158872

[b34] Corbett S. A., Lee L., Wilson C. L. & Schwarzbauer J. E. Covalent cross-linking of fibronectin to fibrin is required for maximal cell adhesion to a fibronectin-fibrin matrix. J Biol Chem 272, 24999–25005 (1997). 9312106 10.1074/jbc.272.40.24999

[b35] Grinnell F., Feld M. & Minter D. Fibroblast adhesion to fibrinogen and fibrin substrata: requirement for cold-insoluble globulin (plasma fibronectin). Cell 19, 517–525 (1980). 7357618 10.1016/0092-8674(80)90526-7

[b36] Corbett S. A., Wilson C. L. & Schwarzbauer J. E. Changes in cell spreading and cytoskeletal organization are induced by adhesion to a fibronectin-fibrin matrix. Blood 88, 158–166 (1996). 8704170

[b37] Peters T. *et al.* Wound-healing defect of CD18(−/−) mice due to a decrease in TGF-beta1 and myofibroblast differentiation. EMBO J. 24, 3400–3410 (2005). 16148944 10.1038/sj.emboj.7600809 PMC1276170

[b38] Lindemann S. *et al.* Activated platelets mediate inflammatory signaling by regulated interleukin 1beta synthesis. J Cell Biol 154, 485–490 (2001). 11489912 10.1083/jcb.200105058 PMC2196422

[b39] Lichtnekert J., Kawakami T., Parks W. C. & Duffield J. S. Changes in macrophage phenotype as the immune response evolves. Curr Opin Pharmacol 13, 555–564 (2013). 23747023 10.1016/j.coph.2013.05.013 PMC3732570

[b40] Midgley A. C. *et al.* Transforming growth factor-β1 (TGF-β1)-stimulated fibroblast to myofibroblast differentiation is mediated by hyaluronan (HA)-facilitated epidermal growth factor receptor (EGFR) and CD44 co-localization in lipid rafts. J. Biol. Chem. 288, 14824–14838 (2013). 23589287 10.1074/jbc.M113.451336 PMC3663506

[b41] Grinnell F. Fibroblasts, Myofibroblasts, and Wound Contraction. J Cell Biol 124, 401–404 (1994). 8106541 10.1083/jcb.124.4.401 PMC2119916

[b42] Bar-Or A. *et al.* Analyses of all matrix metalloproteinase members in leukocytes emphasize monocytes as major inflammatory mediators in multiple sclerosis. Brain 126, 2738–2749 (2003). 14506071 10.1093/brain/awg285

[b43] Zhang Y. *et al.* Disentangling the multifactorial contributions of fibronectin, collagen and cyclic strain on MMP expression and extracellular matrix remodeling by fibroblasts. Matrix Biol. 40, 62–72 (2014). 25217861 10.1016/j.matbio.2014.09.001

[b44] Mirastschijski U. *et al.* Matrix metalloproteinase inhibition delays wound healing and blocks the latent transforming growth factor-beta1-promoted myofibroblast formation and function. Wound Repair Regen 18, 223–234 (2010). 20409148 10.1111/j.1524-475X.2010.00574.x PMC2859473

[b45] Parks W. C., Wilson C. L. & López-Boado Y. S. Matrix metalloproteinases as modulators of inflammation and innate immunity. Nat. Rev. Immunol. 4, 617–629 (2004). 15286728 10.1038/nri1418

[b46] Newby A. C. Metalloproteinase expression in monocytes and macrophages and its relationship to atherosclerotic plaque instability. Arterioscler. Thromb. Vasc. Biol. 28, 2108–2114 (2008). 18772495 10.1161/ATVBAHA.108.173898

[b47] Tonnesen M. G., Feng X. & Clark R. A. Angiogenesis in wound healing. J Investig Dermatol Symp Proc 5, 40–46 (2000). 10.1046/j.1087-0024.2000.00014.x 11147674

[b48] Phelps E. A. & García A. J. Engineering more than a cell: vascularization strategies in tissue engineering. Curr Opin Biotechnol 21, 704–709 (2010). 20638268 10.1016/j.copbio.2010.06.005 PMC2952721

[b49] Lutolf M. P. & Hubbell J. A. Synthetic biomaterials as instructive extracellular microenvironments for morphogenesis in tissue engineering. Nat. Biotechnol. 23, 47–55 (2005). 15637621 10.1038/nbt1055

[b50] Willenborg S. *et al.* CCR2 recruits an inflammatory macrophage subpopulation critical for angiogenesis in tissue repair. Blood 120, 613–625 (2012). 22577176 10.1182/blood-2012-01-403386

[b51] Ferrara N., Gerber H.-P. & LeCouter J. The biology of VEGF and its receptors. Nat Med 9, 669–676 (2003). 12778165 10.1038/nm0603-669

[b52] Gerhardt H. *et al.* VEGF guides angiogenic sprouting utilizing endothelial tip cell filopodia. J Cell Biol 161, 1163–1177 (2003). 12810700 10.1083/jcb.200302047 PMC2172999

[b53] Ballotta V., Smits A. I. P. M., Driessen-Mol A., Bouten C. V. C. & Baaijens F. P. T. Synergistic protein secretion by mesenchymal stromal cells seeded in 3D scaffolds and circulating leukocytes in physiological flow. Biomaterials 35, 9100–9113 (2014). 25112932 10.1016/j.biomaterials.2014.07.042

[b54] Saran U., Gemini Piperni S. & Chatterjee S. Role of angiogenesis in bone repair. Arch Biochem Biophys 561, 109–117 (2014). 25034215 10.1016/j.abb.2014.07.006

[b55] Carano R. A. D. & Filvaroff E. H. Angiogenesis and bone repair. Drug Discov. Today 8, 980–989 (2003). 14643161 10.1016/s1359-6446(03)02866-6

[b56] Hempel U., Hefti T., Dieter P. & Schlottig F. Response of human bone marrow stromal cells, MG-63, and SaOS-2 to titanium-based dental implant surfaces with different topography and surface energy. Clin Oral Implants Res 24, 174–182 (2013). 22092368 10.1111/j.1600-0501.2011.02328.x

[b57] Mustafa Al, M., Agis H., Müller H.-D., Watzek G. & Gruber R. *In vitro* adhesion of fibroblastic cells to titanium alloy discs treated with sodium hydroxide. Clin Oral Implants Res (2013), doi: 10.1111/clr.12294. 24372935

[b58] Bae S. E., Bhang S. H., Kim B.-S. & Park K. Self-assembled extracellular macromolecular matrices and their different osteogenic potential with preosteoblasts and rat bone marrow mesenchymal stromal cells. Biomacromolecules 13, 2811–2820 (2012). 22813212 10.1021/bm300791h

[b59] Tugulu S., Löwe K., Scharnweber D. & Schlottig F. Preparation of superhydrophilic microrough titanium implant surfaces by alkali treatment. J Mater Sci-Mater M 21, 2751–2763 (2010). 20725770 10.1007/s10856-010-4138-x

[b60] Schindelin J. *et al.* Fiji: an open-source platform for biological-image analysis. Nat. Methods 9, 676–682 (2012). 22743772 10.1038/nmeth.2019 PMC3855844

