# Supplementary material for: Synergistic interactions of blood-borne immune cells, fibroblasts and extracellular matrix drive repair in an in vitro peri-implant wound healing model
Source: Sci Rep. 2016 Feb 17;6:21071. doi: 10.1038/srep21071 (PMC4756324; doi:10.1038/srep21071)
Supplement: Supplementary Information [file srep21071-s1.pdf]

Supplementary Information

Synergistic interactions of blood-borne immune cells, fibroblasts and extracellular matrix drive repair in an *in vitro* peri-implant wound healing model

Melanie A Burkhardt, Jasmin Waser, Vincent Milleret, Isabel Gerber, Maximilian Y Emmert, Jasper Foolen, Simon P Hoerstrup, Falko Schlottig, Viola Vogel*

* Corresponding Author: viola.vogel@hest.ethz.ch

Supplementary Figures

Supplementary Figure S1

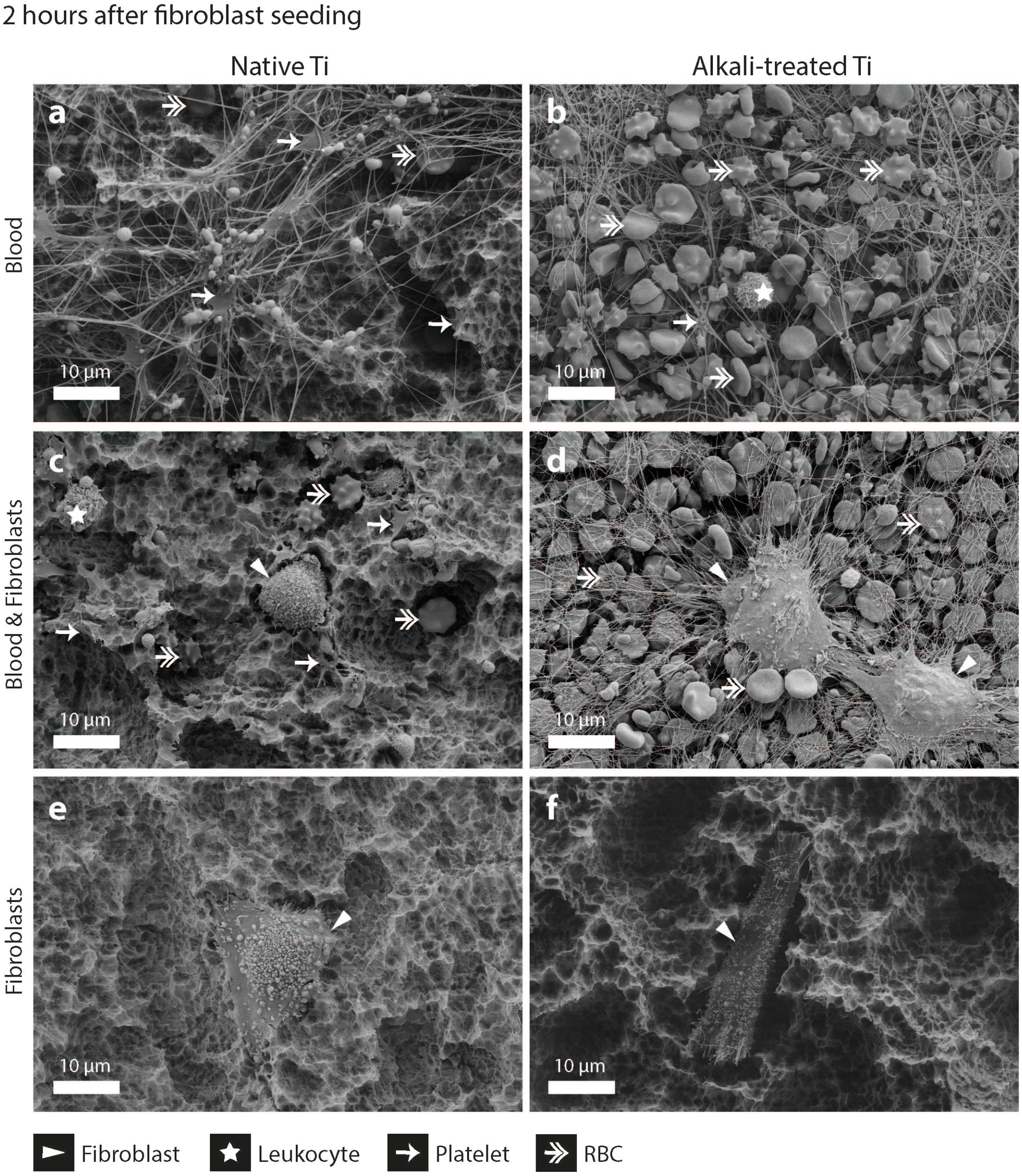

Supplementary Figure S1: Morphology of blood clot and fibroblasts 2 hours after fibroblast seeding. Scanning electron micrographs show morphology of surface-adhering blood clots (a, b), fibroblasts seeded on blood-exposed Ti surfaces (c, d) and fibroblasts alone (e, f) on native (a, c, e) or alkali-treated Ti (b, d, f). Fibroblasts (indicated by an arrowhead) are in the spreading process, guided by ECM on dense blood clots, whereas they attach to Ti surface structures on bare or native blood-exposed Ti. Few blood cells and ECM fibres are found on native Ti, whereas alkali-treated Ti is covered by a thick blood clot after blood exposure. Some blood cells are indicated by stars (leukocytes), arrows (platelets) or double-arrows (red blood cells (RBCs)). Samples were treated as described in Fig. 1c,d.

Supplementary Figure S2

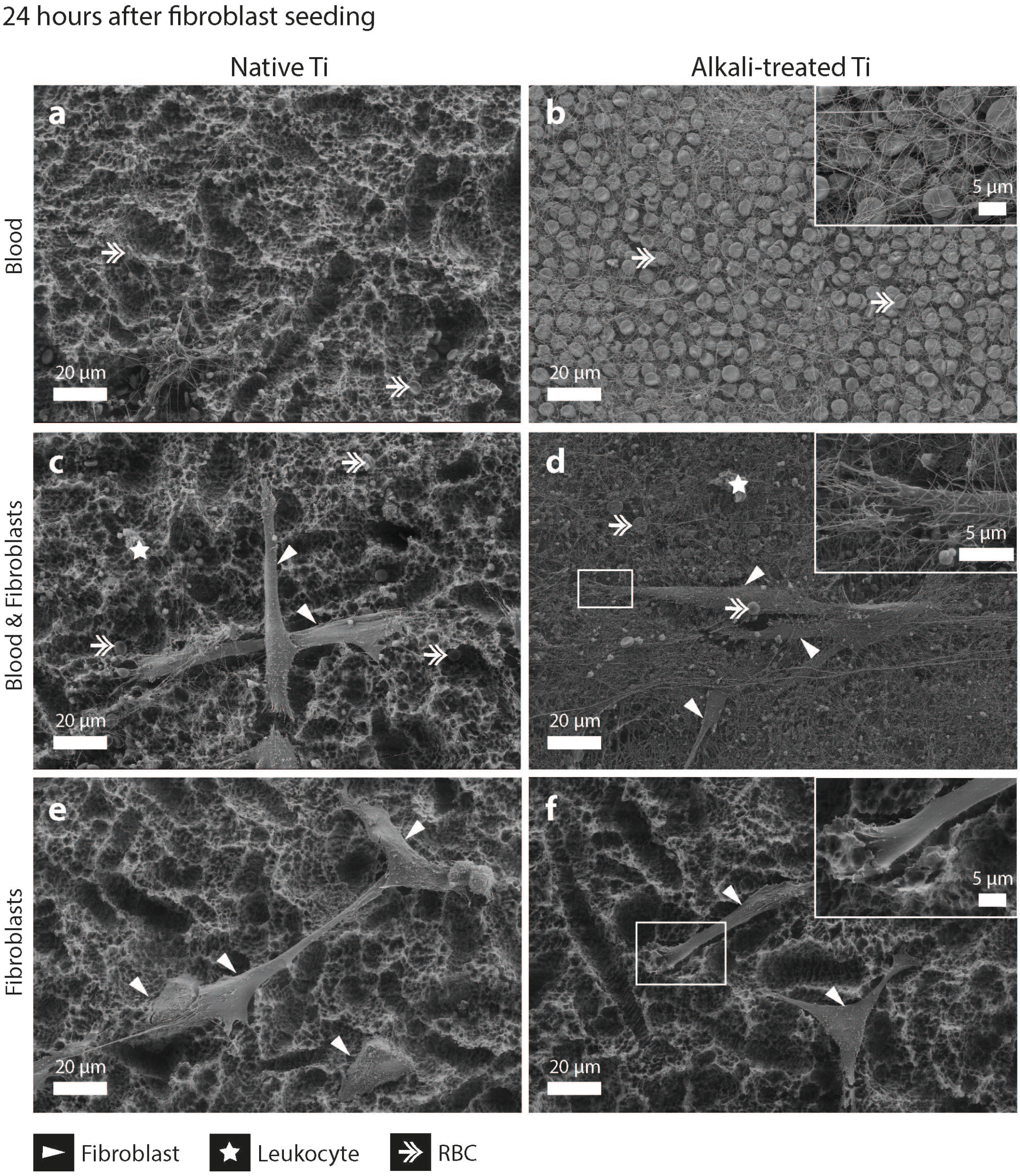

Supplementary Figure S2: Morphology of blood clot and fibroblasts 24 hours after fibroblast seeding. Scanning electron micrographs show morphology of surface-adhering blood clots (a, b), fibroblasts seeded on blood-exposed Ti surfaces (c, d) and fibroblasts alone (e, f) on native (a, c, e) or alkali-treated Ti (b, d, f). Fibroblasts (indicated by an arrowhead) are fully spread after 24 hours and adhering to Ti surface structures or embedded in a dense blood clot surrounded by ECM on alkali-treated Ti. Erythrocytes (RBCs) are indicated with double-arrows, whereas leukocytes are marked with stars. Higher magnification inserts from regions indicated with white boxes (d, f) show dense blood clot morphology (b), fibroblasts interacting with ECM (d) or fibroblasts attaching to rough Ti surface features (f). Samples were treated as described in Fig. 1a,b,e-h.

Supplementary Figure S3

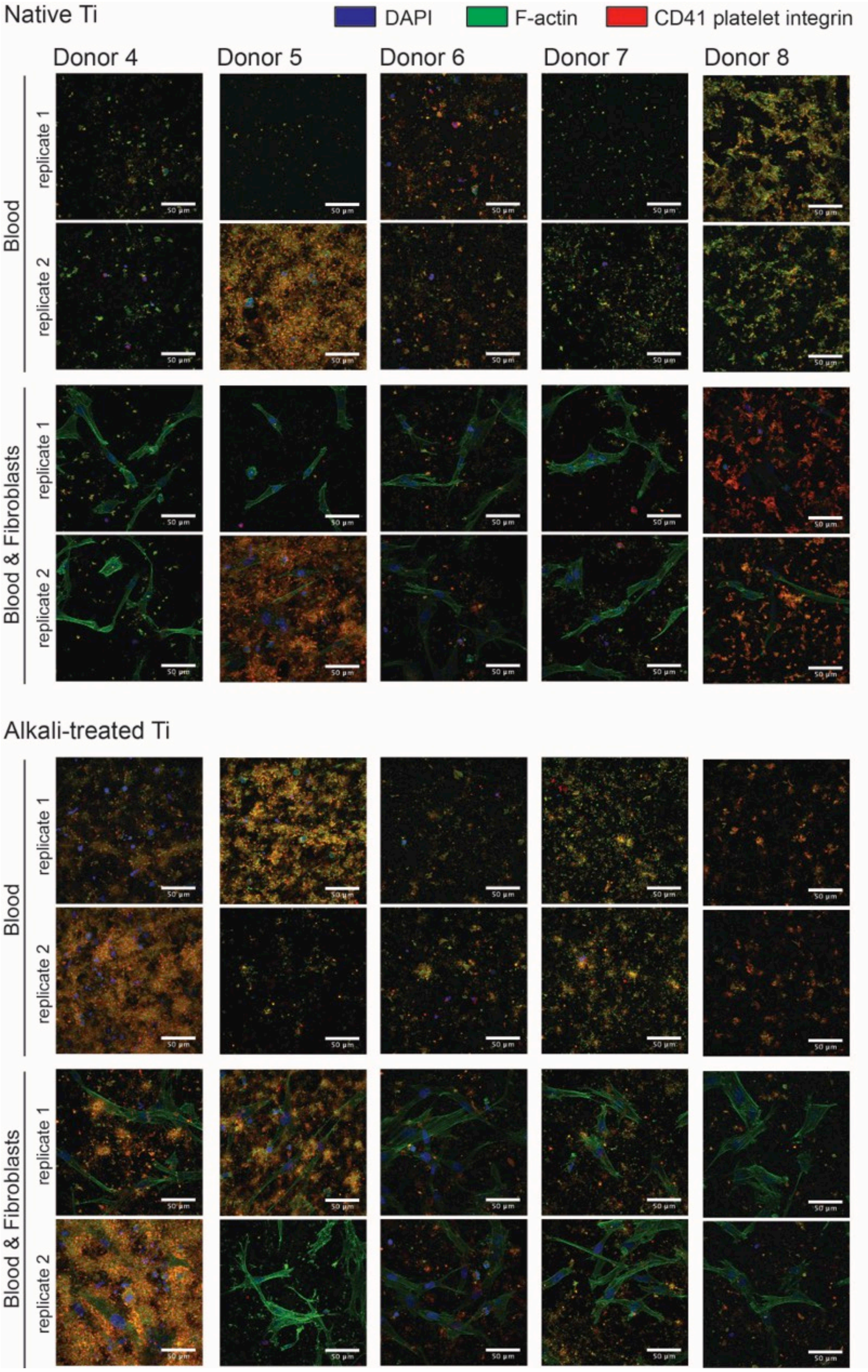

Supplementary Figure S3: Donor to donor variations on replicate samples stained for F-actin, platelets and cell nuclei. Samples were stained for cell nuclei (DAPI) (blue), F-actin (green) and platelet integrin αIIb (CD41) (red). One field of view of all duplicate surfaces of blood only or blood & fibroblast co-culture conditions on native and alkali-treated Ti surfaces from 5 donors are presented as XY maximum intensity projections. Samples were treated as described in Fig. 2b,c,e,f.

Supplementary Figure S4

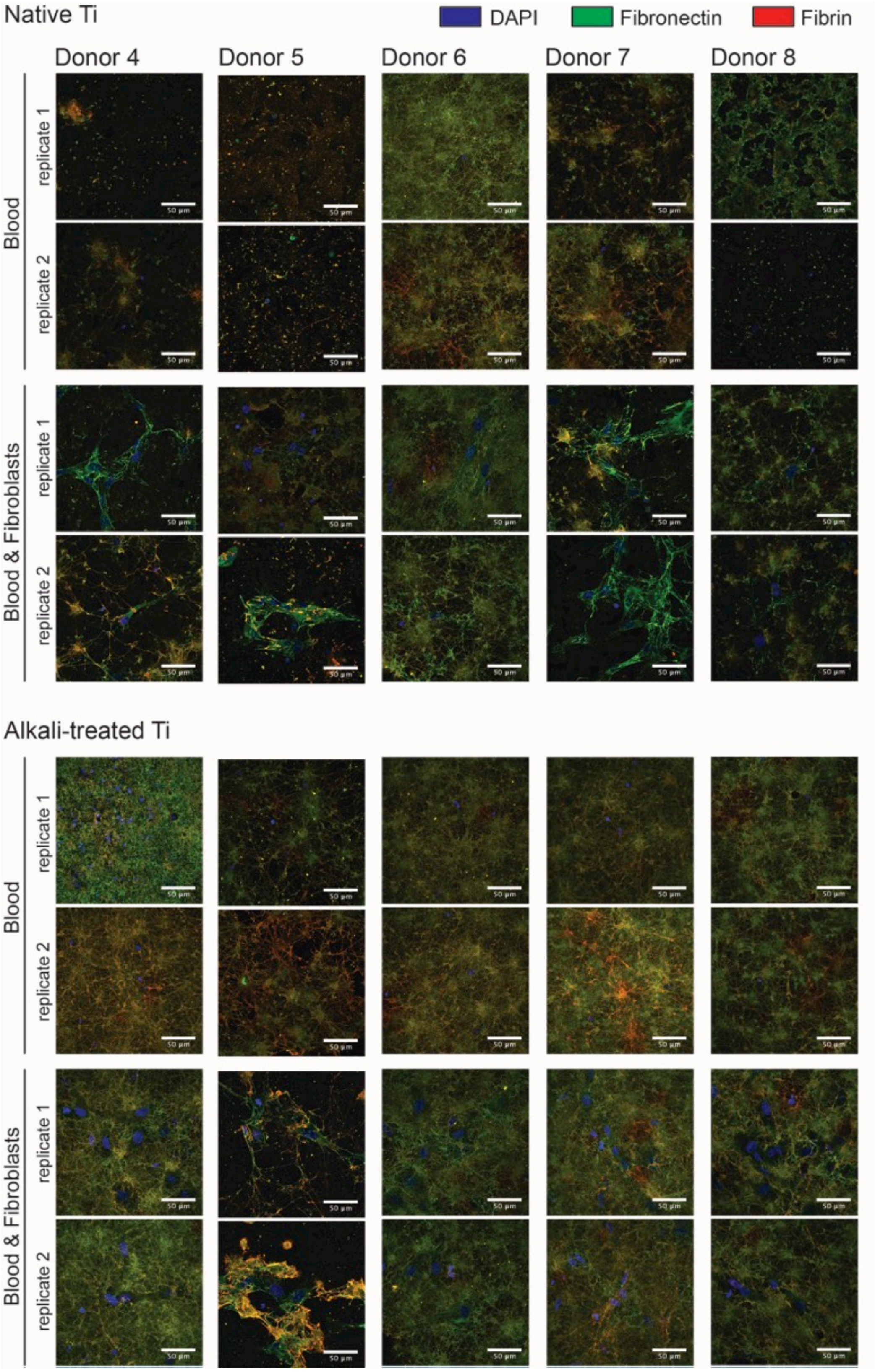

Supplementary Figure S4: Donor to donor variations on replicate samples stained for the ECM components Fn and fibrin. Samples were stained for cell nuclei (DAPI) (blue), Fn (green) and fibrin (red). One field of view of all duplicate surfaces of blood only or blood & fibroblast co-culture conditions on native and alkali-treated Ti surfaces from 5 donors are presented as XY maximum intensity projections. Samples were treated as described in Fig. 4a,b,d,e.

Supplementary Figure S5

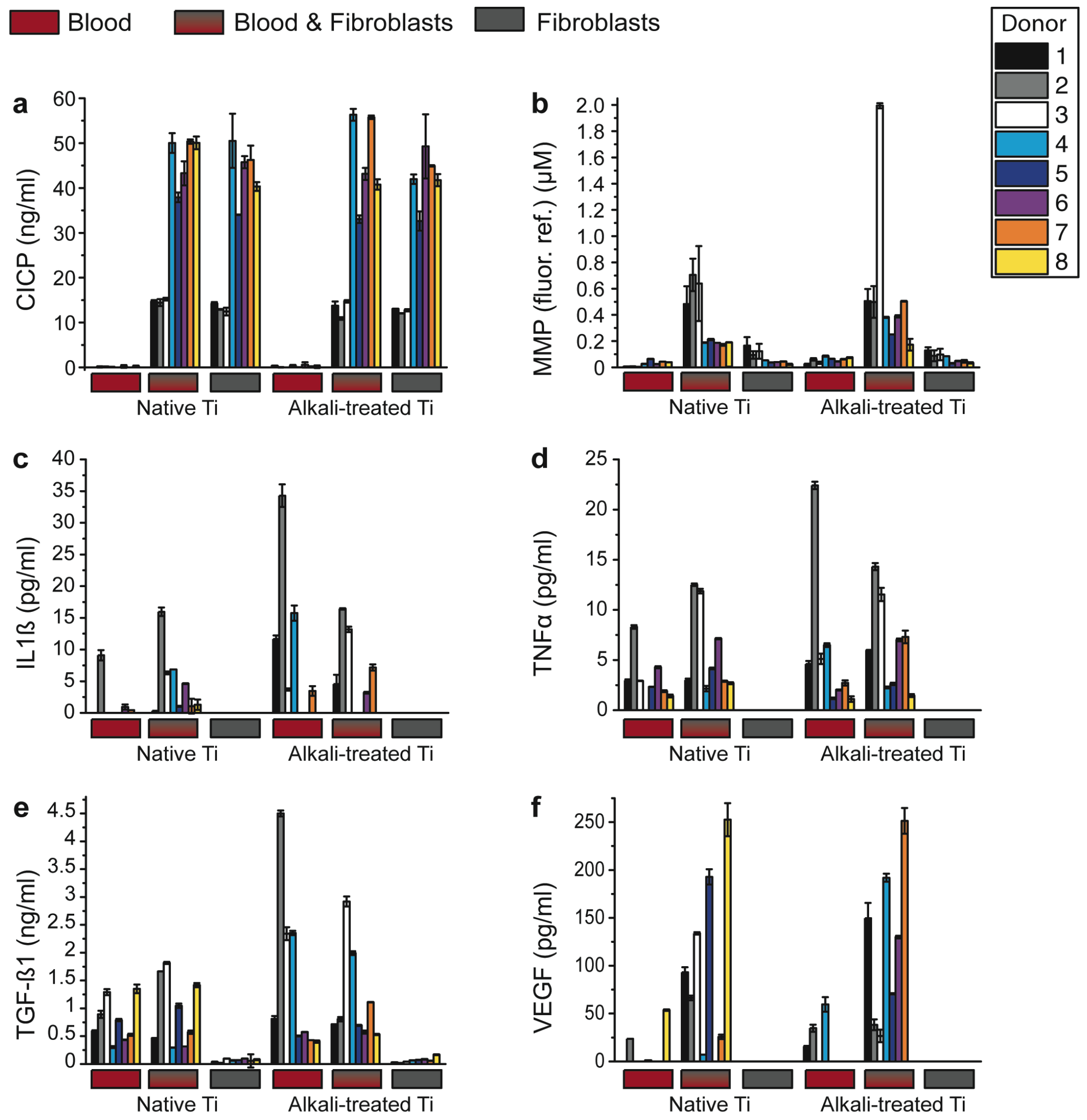

Supplementary Figure S5: Donor-specific concentrations of soluble factors quantified in the supernatants. Concentrations of soluble factors in the supernatants of native and alkali-treated Ti discs exposed to blood (red), blood & fibroblasts (red-grey) and fibroblasts only (grey) 24h after fibroblast seeding are shown for all donors (1-8) as mean values ± standard deviations. Barplots show concentrations of (a) C-terminal peptide of pro-collagen type I (CICP), (b) total matrix metalloproteinase (MMP) including pro-MMP amount standardized to fluorescence reference substrate conversion, (c) IL1β, (d) TNFα, (e) TGF-β1 including latent form and (f) VEGF. Donor averaged values are shown in Fig. 6.
